# Thumb reconstruction by grafting skeletonized amputated phalanges and soft tissue cover – A new technique: A case series

**DOI:** 10.1186/1757-1626-1-22

**Published:** 2008-07-02

**Authors:** Mohammad Murshid Salah, Khalid N Khalid

**Affiliations:** 1 Department of Plastic and Hand Surgery, Hamad Medical Corporation, Doha, Qatar

## Abstract

This study reports five cases of crush-avulsion injury to the thumb at different levels presented at our plastic and hand surgery unit between 2005 and 2007. All of the patients were male labors with machine injuries to the thumb with non-replantable amputations. Distal phalanx or proximal phalanx, or both, were used as a free cortical bone graft. The amputated part was skeletonized keeping the periosteum attached to the cortical bone of the phalanx fixing it to the stump and covering it by either local flap like dorsal metacarpal flap or regional flaps like the distally based pedicled radial forearm flap and neurovascular island sensate flap or groin flap. The results were functionally and cosmetically good and follow up X rays showed no osteoporotic resorption after one year.

## Introduction

The function of the thumb is critical to overall hand function, uniquely endowed with anatomical features that allow circumduction and apposition. The thumb is the most important digit for the pinch and grasp function of the hand. Indeed it contributes approximately 40% of hand function. Therefore, every effort should be done to replant or reconstruct amputated thumbs to regain hand function [1,2]. Although development in microsurgical techniques changes the strategy of management of thumb amputations other modalities of reconstruction of non-replantable amputations still working. Factors taken in consideration in selecting surgical options include: age, sex, occupational demands, hand dominance, mechanism of injury, condition of the amputated part and objective needs of the patient. The functional requirements of the thumb are adequate sensibility, sufficient length and mobility, freedom from pain. By all means skill and experience of the surgeon is required. For reconstructive purposes the thumb is divided into three zones;

Zone one: up to interphalangeal joint.

Zone two: up to neck of metacarpal bone.

Zone Three: up to the Carpometacarpal Joint.

Tip injuries are managed either conservatively by secondary intention, skin graft, V-Y flap, lateral triangular advancement flap, palmer advancement (Moberg flap), cross finger flap, dorsal metacarpal Foucher flap or neurovascular island flaps (Littler) [3].

For more proximal amputation i.e. zone two and three; replantation if possible is the best way of management [4], otherwise either phalangization of the metacarpal bone by deepening of the first web using Z plasty, four-flap plasty, distractive lengthening of the first metacarpal bone [5-7], or pollicization of index stump or finger [8]. Osteoplastic reconstruction using composite osteofasciocutaneous groin or radial forearm flap still useful way. Distally based island and free osteocutaneous flaps provide vascularised bone reconstruction which achieves rapid bone union, undergoes little resorption and shows good infection resisting capabilities [9]. Free toe or pulp transfer still very excellent way if feasible but not all patients accept such procedure and it is demanding [10,11]. We start using skeletonized phalanx of the amputated part as a free cortical bone graft with its periosteum which give us perfect skeleton instead of taking ileac or radial bone and cover it with soft tissue. We assume that the periosteum as a free graft start taking blood supply from the surrounding soft tissue and then it give nourishment to the cortical bone this is shown in the callus formed at the fracture site and ossification and complete union later on.

## Case presentation

From January 2005 to January 2007 we have done five cases in our hand and plastic surgery unit (Table 1). All of them are labors with age between 22–35 years sustained work traumas with avulsion crush injuries to thumb. In all of them the amputated thumb was not replantable. All patients have now returned to work.

**Table 1:** Clinical data, presentation, management and outcome of 5 cases of thumb avulsion and crush injury

#	Gender/age	Occupation	Injured thumb	Mechanism	Level of amputation	Phalanx used	Soft tissue	Matching with other hand	MCP/ROM	Sensation	Grip & pinch	Duration of Treatment
1	M/22 y	Tire repairing	left	Crush/avulsion	Base of proximal phalanx	Proximal and distal of same thumb	Distally based fasciocutaneous RFF	Good	0 – 45	Protective	25 kg 5 kg	3 months
2	M/25 y	Mechanic	right	Avulsion	IP joint and soft tissue at MCP joint	Distal phalanx of same hand	Distally based fasciocutaneous RFF	Good	0 – 50	Protective	27 kg 7 kg	2 months
3	M/27 y	Steel fitter	left	Saw cut	Base of prox. Phalanx with loss of dorsal soft tissue	Distal and middle phalanx of amputated index	Proximally based dorsal metacarpal flap	Acceptable	0 – 50	Normal volar sensation & dorsal protective	30 kg 8 kg	3 months
4	M/24 y	Mechanic	right	Car dropped on his hand	Comminuted lost segments of proximal and distal phalanx	Middle and distal phalanx of amputated index	Same soft tissue and skin graft	Very good	0 – 55	Normal	35 kg 10 kg	4 months
5	M/32 y	Carpenter	right	Saw amputation on 2 levels	MCP joint and IP joints	Proximal ph. of same thumb	Groin flap	Reasonable	0 – 35	Protective	20 kg 5 kg	5 months

RFF: Radial Forearm Flap

IP: Intraphalangeal

MCP: Metacarpal phalangeal.

### Case One

This is 22 years old right-handed male labor his left thumb was caught by tire puncture repairing machine sustained crush avulsion injury with the skin and soft tissue crushed and avulsed at the level of base of the metacarpal bone and the bone at the base of the proximal phalanx and tendons at the tenomuscular junction and the neurovascular bundle avulsed very distally. It was not replantable.

**Figure 1:**
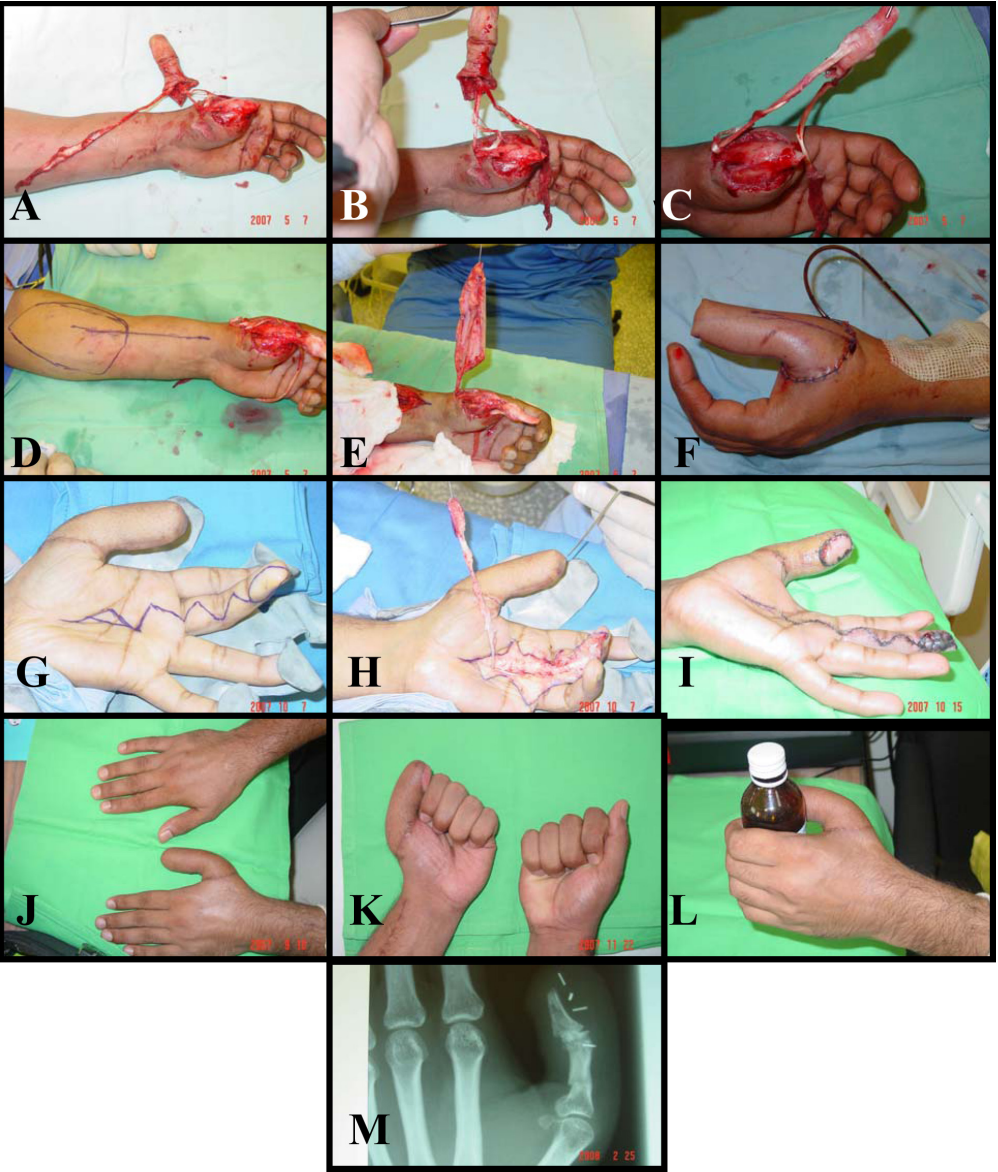
A, B crush avulsion injury. C: Skelotinize the bone. D: Marking the radial forearm flap & fixing the bone graft by two crossing K wires. E, F: Raising & setting the flap. G, H, I: Marking raising & setting the sensate island (Littler flap). J, K, L: final result M: X-ray shows bone healing.

We discuss the plan of management with him but he refused to sacrifice his index or toe for pollicization or free toe transfer so we obtained consent from him for reconstruction. In theatre, we skeletinized the amputated part leaving the periostium and ligaments attached to the bones fixing it back by two crossing K wires, tendons repaired to muscle, the bone was covered by a distally based fasciocutaneous radial forearm flap and the donor site covered with split thickness skin graft. Later neurovascular island flap taken from the ulnar side of the long finger distal phalanx to give sensation to the reconstructed thumb. The patient regain good range of motion through the preserved metacarpophalangeal joint and carpometacarpal joint. After three months he was back to work (Figure 1).

### Case Two

**Figure 2:**
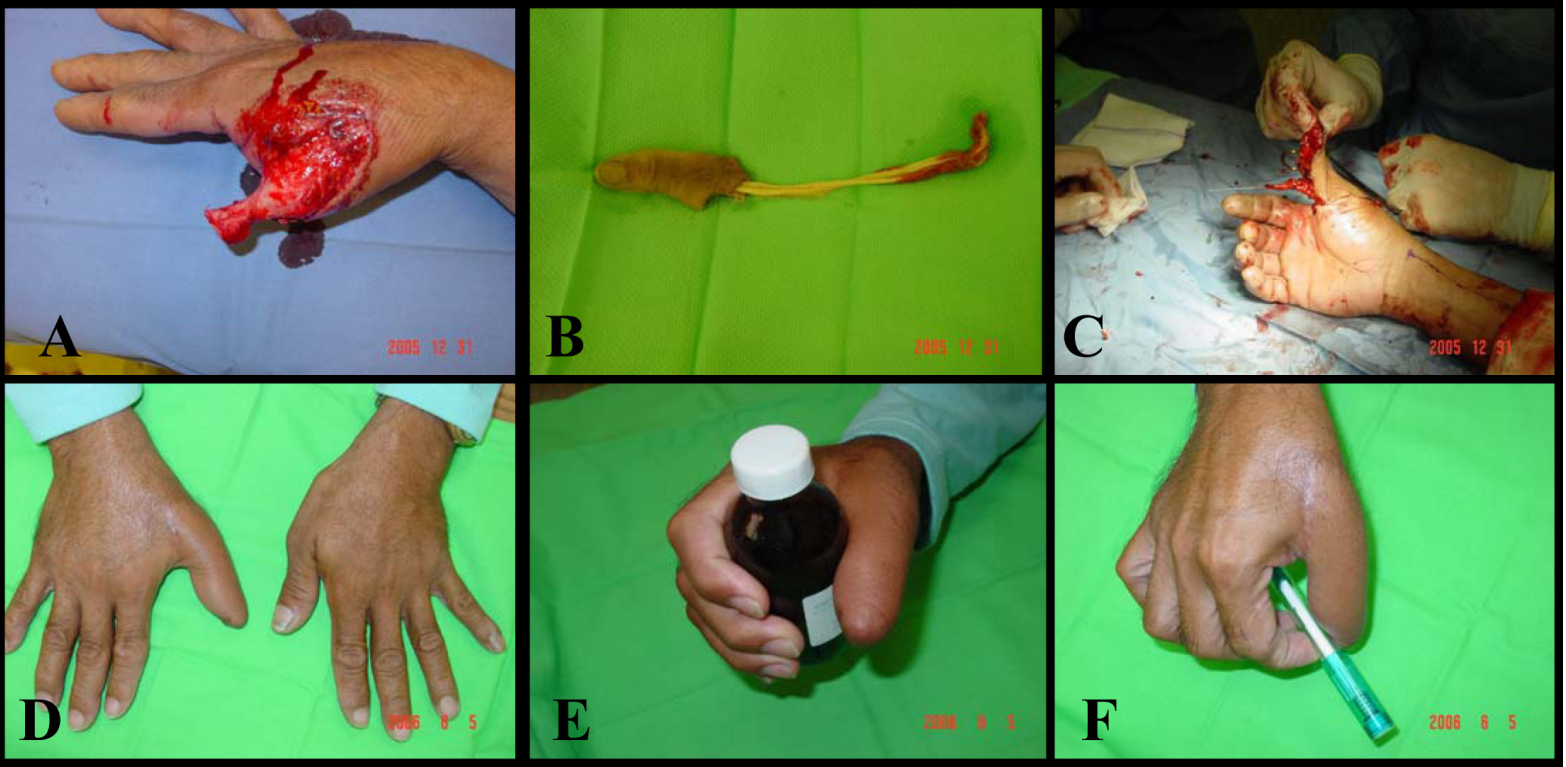
A, B: Avulsion of bone at interphalangeal joint & soft tissue at the metacarpophalangeal joint. C: the bone skeletonized & fixed to the stump & covered with distally based radial forearm fasciocutaneous flap. D, E, F; Final functional & cosmetic result.

This was a 25 year-old right-handed male mechanic. His right thumb was trapped and crushed between two hard objects causing avulsion of skin and soft tissue at the MCP joint. The bone avulsed at the interphalangeal joint with the extensor and flexor tendons attached to it and detached from its muscolotendonous junction. Same reconstruction procedures done as shown (Figure 2).

### Case Three

**Figure 3:**
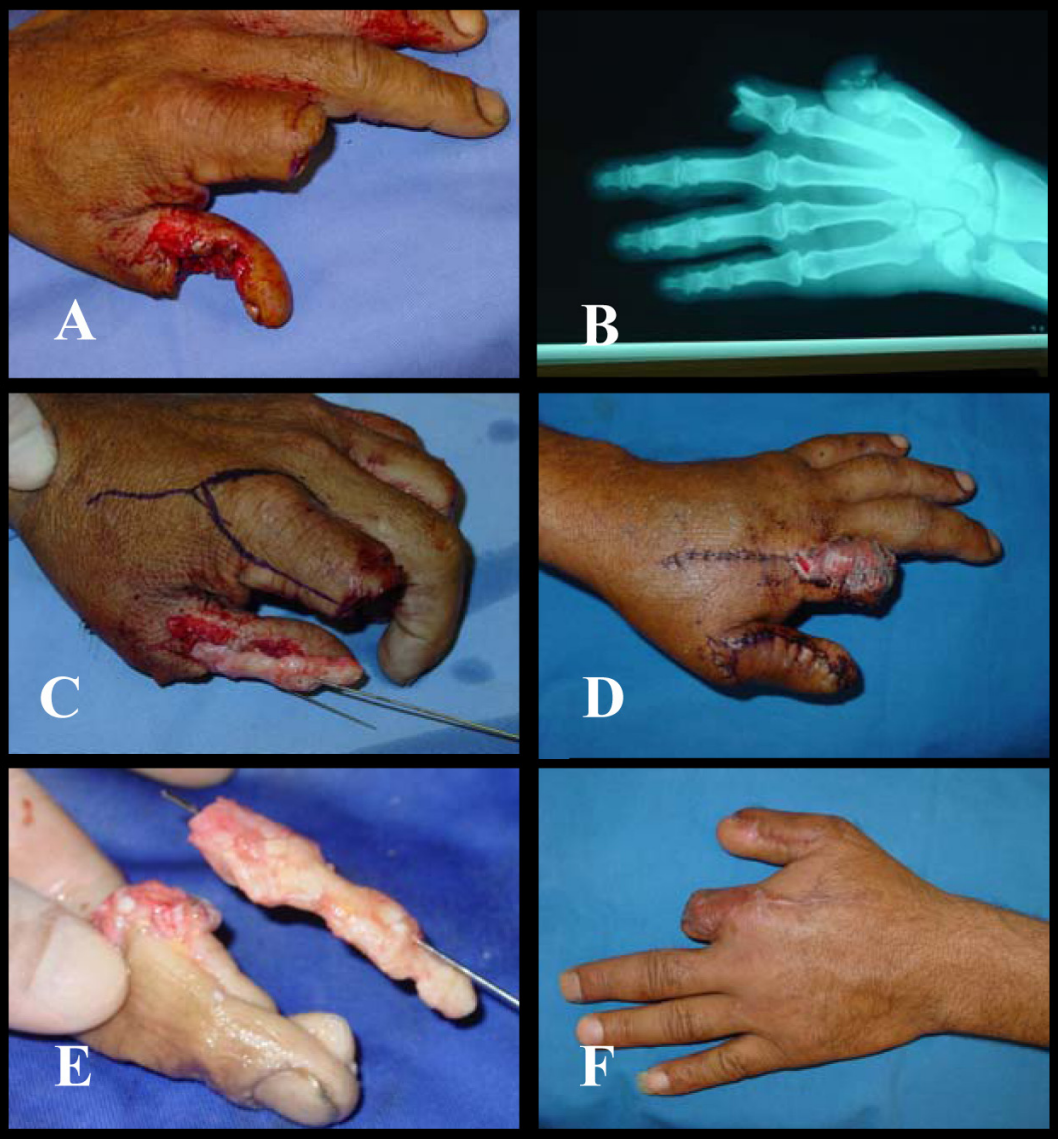
A, B: Electric saw injury with amputation of the index at the proximal interphalangeal joint level & loss of the dorsal skin & bone of the thumb. C: the bone of the index used to build the skeleton of the thumb. D, E: proximally based 2^nd ^dorsal metacarpal flap used as a dorsal soft tissue cover. F: the final result.

This was a 27 year-old-right-handed male steel fitter, his left hand was cut by an electric saw sustained amputation of the index finger at the proximal interphalangeal joint and splitting of the thumb longitudinally with tangential loss of dorsal skin with soft tissue up to the base of the proximal phalanx. The metacarpophalangeal joint still intact. The volar skin with both neurovascular bundles was intact. So we skeletonize the amputated part of index and used as free cortical bone graft to build up the skeletal structure of the thumb and covered by proximally based 2^nd ^dorsal metacarpal artery (Foucher flap) (Figure 3).

### Case Four

**Figure 4:**
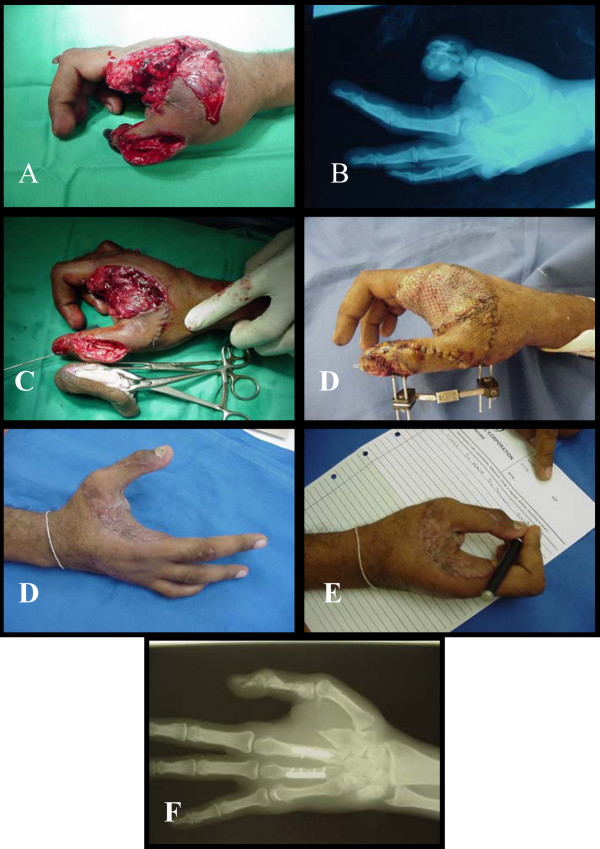
A, B: Injury with crush amputation of the second ray & comminution with bone loss of the thumbs phalanges. C: Phalanx of the index used as a free graft to build the thumb skeleton. D: Soft tissue cover. E, F, G: final result.

This was a 24-year-old right-handed male mechanic. A car dropped on his right hand sustained severe crush injury and total amputation of the 2^nd ^ray at the neck of the 2^nd ^metacarpal bone. The thumb was badly crushed and burst, the bone is severely comminuted so we took cortical bone graft from the index to reconstruct the thumb skeleton and hold by external fixator. The raw area covered by split thickness skin graft (Figure 4).

### Case Five

**Figure 5:**
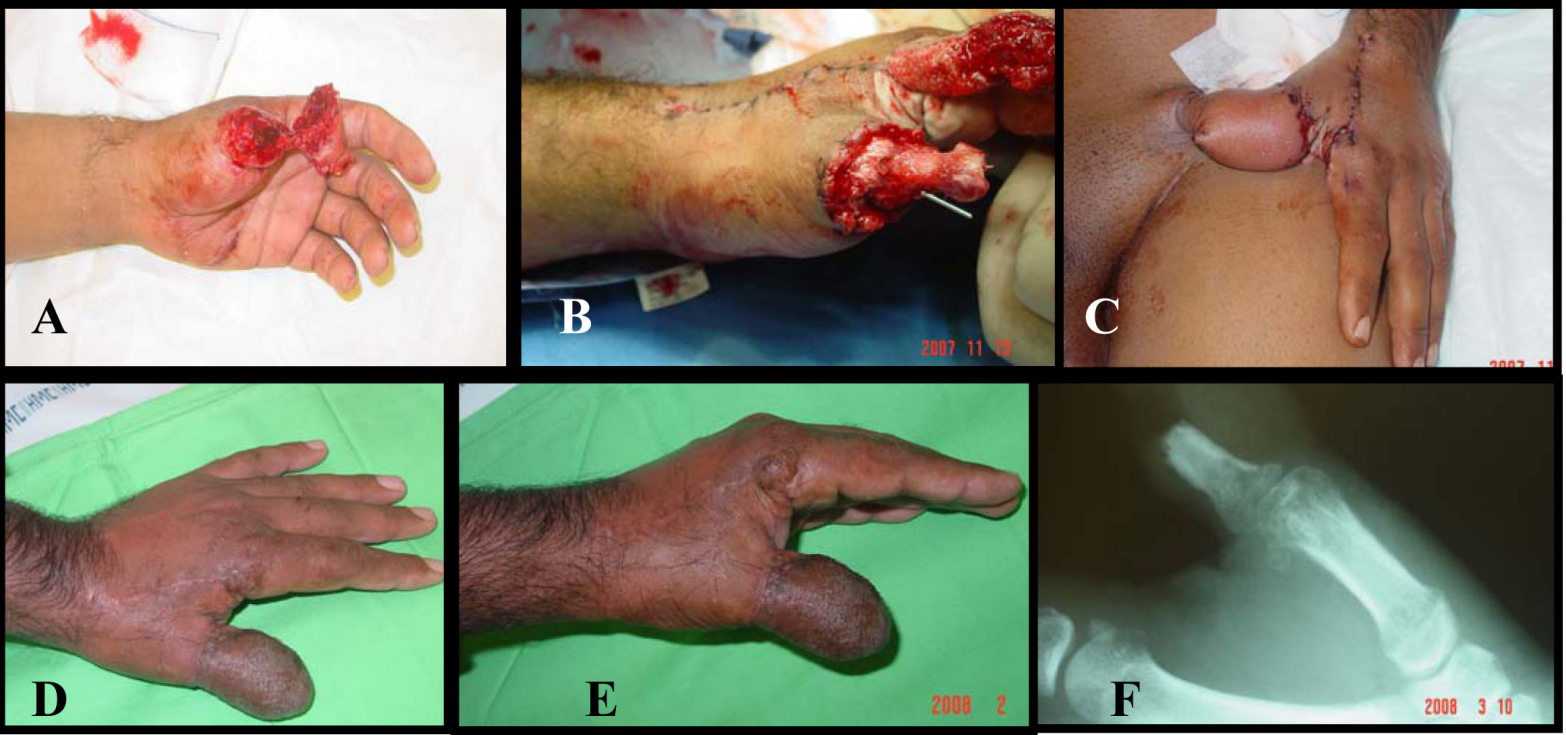
A: the amputation at two levels. B: Fixing the cortical bone graft. C: soft tissue cover by groin flap. D: division of the flap. E: final result. F: x-ray shows the bone healing.

This was a 32 year old right handed male carpenter his right thumb was cut by an electric saw sustained amputation at the base of the proximal phalanx and another amputation at the IP joint. Skeletinization and fixation of the bone and coverage by groin flap (Figure 5).

## Discussion

As the thumb is the most important digit for hand function it should by any means replanted if possible or reconstructed if not. The reconstruction of posttraumatic thumb defects is a challenging and rewarding surgical endeavour. The value of a functioning thumb is immense, and its reconstruction is worthy of considerable effort. Despite the elegant reconstructive options available, the best results are obtained with replantation or revascularization whenever possible. Finally, the treatment plan always must be derived from a careful assessment of each patient's posttraumatic function and specific reconstructive needs. There are many ways to do so. Age, sex, profession of the patient hand dominance, mechanism of injury, level of amputation, experience of the surgeon, all play important role in decision making for the way of reconstruction. One way of reconstruction is to replant the cortical bone of the amputated part as a free graft. It was well known that the bone should be either cancellus or corticocancellus to be taken as a free graft or vascularized as a pedicled or free osteofasciocutaneous flap, but these operations have risk of fracture donor bone in addition to the need for a second painful operative site. To take a cortical bone as a free graft in thumb reconstruction can provide good skeletal support with the same contour as the non-amputated thumb with no sacrifice to other bones. This is covered by thin flap like the radial forearm flap we prefer this flap because it is thin, pliable and sizable, though it leaves donor site morbidity. While groin flap is a bit bulky flap with less donor site morbidity. The idea of using cortical bone as free graft came from the use of same technique in symbrachydactyly where we use toe phalanx to build the nubbin skeleton. We assume that the periostum, which is left attached to the bone, first pick up its blood supply from the surrounding soft tissue then it provide the cortical bone with blood. We confirm this by exploring the bone for sensate flap cover we found blood coming from the medulla of the bone, secondly the two bones healed by callus formation fixing the fracture site and thirdly no bone resorption by x-ray after one year.

## Conclusion

After a severe digital or extremity injury, the replantation surgeon should always seek to make the best use out of what tissue is available for reconstruction. Exercising sound surgical judgment and being creative allow the surgeon to restore function to critical areas of the hand or extremity by the judicious use of available tissues that would otherwise be discarded. The use of spare parts should, therefore, always be considered to facilitate digital or extremity reconstruction when routine replantation is not possible or is likely to produce a poor functional result. The surgeon should always try to use available non replantable tissue to preserve length, obtain soft tissue coverage, or most importantly improve the function of remaining less injured digits. We found in this study the use of amputated phalanx as free graft one interesting and useful way of thumb reconstruction.

## Consent

Written informed consent was obtained from all the patients for publication of this case report and accompanying images. Copies of the written consent are available for review by the Editor-in-Chief of this journal.
